# Semantic Congruency Modulates the Effect of Attentional Load on the Audiovisual Integration of Animate Images and Sounds

**DOI:** 10.1177/2041669520981096

**Published:** 2020-12-31

**Authors:** Qingqing Li, Qiong Wu, Yiyang Yu, Fengxia Wu, Satoshi Takahashi, Yoshimichi Ejima, Jiajia Yang, Jinglong Wu

**Affiliations:** Cognitive Neuroscience Laboratory, Graduate School of Natural Science and Technology, Okayama University, Okayama, Japan; Department of Psychology, Suzhou University of Science and Technology, Suzhou, China; Cognitive Neuroscience Laboratory, Graduate School of Natural Science and Technology, Okayama University, Okayama, Japan; Cognitive Neuroscience Laboratory, Graduate School of Interdisciplinary Science and Engineering in Health Systems, Okayama University, Okayama, Japan; Cognitive Neuroscience Laboratory, Graduate School of Interdisciplinary Science and Engineering in Health Systems, Okayama University, Okayama, Japan; Beijing Advanced Innovation Center for Intelligent Robots and Systems, Beijing Institute of Technology, Beijing, China

**Keywords:** audiovisual integration, common object, attentional load, semantic congruency, dual-task paradigm

## Abstract

Attentional processes play a complex and multifaceted role in the integration of input from different sensory modalities. However, whether increased attentional load disrupts the audiovisual (AV) integration of common objects that involve semantic content remains unclear. Furthermore, knowledge regarding how semantic congruency interacts with attentional load to influence the AV integration of common objects is limited. We investigated these questions by examining AV integration under various attentional-load conditions. AV integration was assessed by adopting an animal identification task using unisensory (animal images and sounds) and AV stimuli (semantically congruent AV objects and semantically incongruent AV objects), while attentional load was manipulated by using a rapid serial visual presentation task. Our results indicate that attentional load did not attenuate the integration of semantically congruent AV objects. However, semantically incongruent animal sounds and images were not integrated (as there was no multisensory facilitation), and the interference effect produced by the semantically incongruent AV objects was reduced by increased attentional-load manipulations. These findings highlight the critical role of semantic congruency in modulating the effect of attentional load on the AV integration of common objects.

In daily life, individuals usually receive information from many sensory modalities, and the human brain can combine and bind the available information from multiple senses to better perceive the external environment. The phenomenon by which stimuli from multiple sensory organs can be integrated into a coherent representation to better perceive information is called multisensory integration (Giard & Peronnet, 1999; Stein et al., 2010). Multisensory integration has been demonstrated to occur in several different brain areas at different stages of sensory processing using different stimulus types (Koelewijn et al., 2010). For example, the multisensory integration of complex stimuli, especially high-level semantic stimuli, likely occurs at higher cortical areas (Doehrmann & Naumer, 2008; Koelewijn et al., 2010). Moreover, the typical associations between complex auditory and visual stimuli at the level of semantic content rely on the audiovisual (AV) integration of common objects (Doehrmann & Naumer, 2008; Hein et al., 2007).

The AV integration of common objects, which involves interactions between a complex visual stimulus and a sound counterpart of living and nonliving familiar objects, such as the binding of the picture of a dog and a corresponding barking sound, closely corresponds to semantic content and operates on a higher level (Doehrmann & Naumer, 2008; Taylor et al., 2006). Moreover, recent studies have shown that semantic congruency, which modulates the semantic association between the individual sensory elements of a single object (Noppeney et al., 2008), has an impact on AV integration. Specifically, bimodal stimuli conveying semantically congruent information can be preferentially selected to improve behavioural performance, whereas incongruent bimodal stimuli impair performance (Molholm et al., 2004; Suied et al., 2009). At the neural level, it has been reported that the integration of semantically congruent AV combinations of common objects evokes stronger activations of posterior temporal regions around the superior temporal sulcus and middle temporal gyrus than incongruent combinations (Beauchamp et al., 2004). Furthermore, the processing of semantic congruency between the unimodal components of a multisensory signal involves higher level cognitive processing, and semantic congruency was proposed as a factor that determines the extent of attentional effects on AV integration (Mishra & Gazzaley, 2012; Molholm et al., 2007; Mozolic et al., 2008; Zimmer et al., 2010). Specifically, using spoken and written nouns in a target detection task, Mishra and Gazzaley (2012) showed that compared with selective attention to either the visual or the auditory modality, distributing attention across both auditory and visual domains enhances performance for congruent AV stimuli but resolves interference for incongruent AV stimuli. Thus, it seems that the integration of semantically congruent AV stimuli may be less susceptible to top-down attentional controls than the interference effect of incongruent AV stimuli.

In fact, the role of attention in the integration of input from different sensory modalities is complex and multifaceted (Macaluso et al., 2016; Talsma et al., 2010), and whether the occurrence of multisensory integration is relatively automatic and not affected by top-down attentional control has become an ongoing debate (Hartcher-O’Brien et al., 2017; Talsma, 2015). Currently, many studies are beginning to use a dual-task paradigm in which a distracter task is adopted to modulate the levels of the endogenous attentional resources available for the secondary task to explore the effects of attentional load on multisensory integration processing. Using this approach, it has been demonstrated that the “ventriloquist effect” (the temporal integration of simple AV stimuli) is not influenced by attentional load. Specifically, a shift in auditory localization toward peripheral flashes can still be found regardless of whether attention was exogenously directed away from the flashes (Vroomen et al., 2001). In a similar manner, some findings indicate that multisensory cues can more effectively attract spatial attention even under high attentional load than unimodal cues, indicating that the spatial integration of simple multisensory cues is not affected by increased attentional demands (Ho et al., 2009; Santangelo & Spence, 2007). In contrast, some results have demonstrated that attentional load severely interfered with AV speech integration as indexed by the McGurk effect, in which a speech sound paired with an incongruent lip movement leads to a fused speech sound (Alsius et al., 2005, 2007; Gibney et al., 2017); this type of speech perception is usually considered highly complex and requires extensive neural processing (Cappa, 2016). Nevertheless, although these studies have obtained contradictory experimental findings, they investigated different aspects of multisensory integration (temporal or spatial integration of simple multisensory stimuli; AV speech perception). Furthermore, it seems that several aspects related to the impact of attentional load on multisensory integration have not been fully studied; specifically, it remains an open question whether attentional load also disrupts AV integration of common objects. Moreover, how semantic congruency interacts with attentional load to influence the AV integration of common objects also remains unclear.

Thus, the purpose of the current study was to apply a dual-task paradigm to rigorously examine how semantic congruency interacts with attentional load to influence the AV integration of common objects. The dual-task paradigm reduces the attentional capacity dedicated to the main task because dividing attention between two concurrent tasks results in a decrease in behavioural performance relative to when only the main task is performed (Abernethy, 1988; Plummer & Eskes, 2015). In addition, a distractor task of low difficulty allows the allocation of spare attentional resources to another simultaneous task; however, performing a highly difficult distractor task may exhaust the attentional resources that can be allocated to another task (Lavie, 2005, 2010). Thus, by increasing the difficulty of the distractor task, attentional load can be controlled at different levels. We adopted a rapid serial visual presentation (RSVP) task as the distractor task to impose different levels of attentional load, namely, no load, low load, and high load. In addition, we also controlled the semantic congruency in the AV integration task by adopting semantically congruent AV objects (e.g., dogs with barks) and semantically incongruent AV objects (e.g., birds with barks) of common objects. Finally, our hypotheses were as follows: (a) The integration of semantically congruent AV object features would not be significantly attenuated by increased attentional load; (b) however, the multisensory interference effect of semantically incongruent AV object features would be significantly decreased by increased attentional load. Our behavioural results are evaluated from the perspective of these hypotheses.

## Methods

### Participants

A total of 20 volunteers (5 females, mean age of 25 years) participated in this study. The participants reported normal or corrected-to-normal hearing and vision. All participants provided written informed consent, and the study procedures were approved in advance by the ethics committee of Okayama University. Two participants were excluded from further analyses due to poor data quality, specifically because they had low average accuracy of the AV integration task even under the no-load condition (70% accuracy). Therefore, data from 18 subjects were analysed (4 females; mean age 26 years, ranging from 18 to 31 years).

### Apparatus and Materials

All study procedures were completed in a dimly lit, electrically shielded, and sound-attenuated room, specifically, a laboratory room at Okayama University, Japan. Each participant positioned his or her head on a chin rest. All visual stimuli were presented on a 24-inch VG 248LCD monitor (made by ASUS, Taiwan) with a screen resolution of 1,920 × 1,080 and a refresh rate of 144 Hz set at a viewing distance of 57 cm from the participant. Auditory stimuli were presented through speakers located on the central monitor. In addition, two speakers (Harman/Kardon HK206, frequency response: 90–20,000 Hz) were used to present the auditory stimuli. MATLAB software (R2014b, MathWorks, MA, Psychtoolbox-3) was used to present the experimental stimuli and record the participants’ responses.

We administered the animal identification task (AV integration task) with the following four basic stimulus types, each presented with equal probability: (a) sounds alone, (b) pictures alone, (c) paired pictures and sounds belonging to the same animal, and (d) paired pictures and sounds belonging to different animals. The images included line drawings of a dog, bee, frog, bird, and pig developed by the Snodgrass and Vanderwart set (Snodgrass & Vanderwart, 1980) and were standardized by familiarity and complexity. All visual stimuli were presented on the lower left or lower right quadrant of the screen for 300 ms (subtending a 12° visual angle to the left or right of the centre and a 5° angle below the central fixation point).

**Figure 1.**
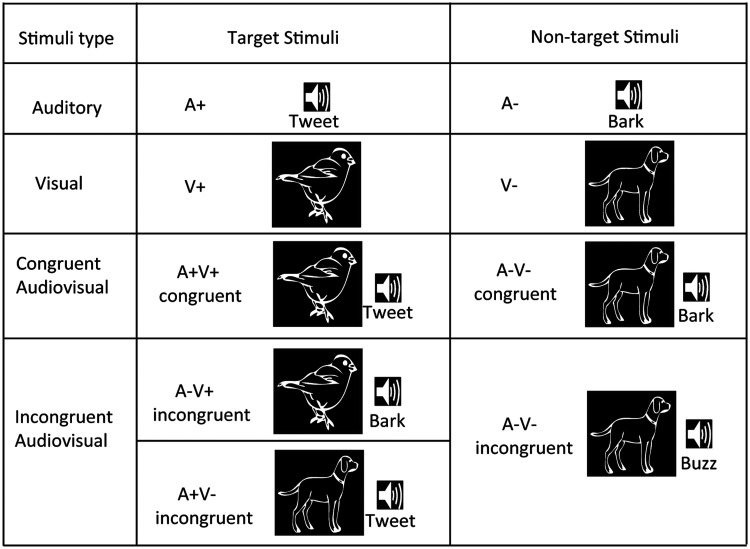
The stimulus types used in the animal identification tasks. In this study, five target stimulus types and four nontarget stimulus types were derived from the four basic stimulus types. The stimulus under each type is just one example.

The sounds of these five animals were collected through internet searches (http://sc.chinaz.com/tag_yinxiao/DongWuJiaoSheng.html) and later standardized and modified such that each single animal sound had a duration of 300 ms. The animal sounds were presented at a comfortable listening level of the ∼75 dB sound pressure level. Furthermore, in addition to the unimodal stimuli (animal pictures alone and animal sounds alone), the pictures and sounds of animals were also combined to form both congruent pairs (combinations of pictures and sounds belonging to the same animal) and incongruent pairs (combinations of pictures and sounds belonging to different animals). Of note, the images or sounds of a “bird” served as the target stimuli. Participants were asked to react as fast as possible to a target object (“bird”) presented in the visual and/or auditory modality and to inhibit a distractor object (go/no go task). It was further explained to them that they also had to respond to semantically incongruent stimuli, in which only the visual or the auditory element was the target. Finally, five target stimulus types and four nontarget stimulus types were derived from the four basic stimulus types (Figure 1). The five target stimulus types were as follows: visual target (V+, a picture of a bird), auditory target (A+, the tweet of a bird), a picture and sound pair in which both were targets (V + A+, a picture of a bird and the tweet of a bird), a picture and sound pair in which only the picture was a target (A−V+; e.g., a picture of a bird and the bark of a dog), and a picture and sound pair in which only the sound was a target (A + V−; e.g., a picture of a dog and the tweet of a bird).

The four nontarget stimulus types were as follows: an animal picture (V−), an animal sound (A−), a paired picture and sound of the same animal (congruent A−V−), and a picture of one animal paired with the sound of another animal (incongruent V−A−). Thus, there were nine total trial types (five target stimulus types: V+, A+, V + A+, A−V+, and A + V− and four nontarget stimulus types: V−, A−, Congruent A−V−, and Incongruent A−V−), presentation of these stimulus types were equiprobable, and there was 64 trials with each stimulus type. Therefore, a total of 576 trials were included under each attentional-load condition in the experiment. To avoid the fatigue, these trials were divided into 4 main blocks of 144 trials each under each load condition.

**Figure 2.**
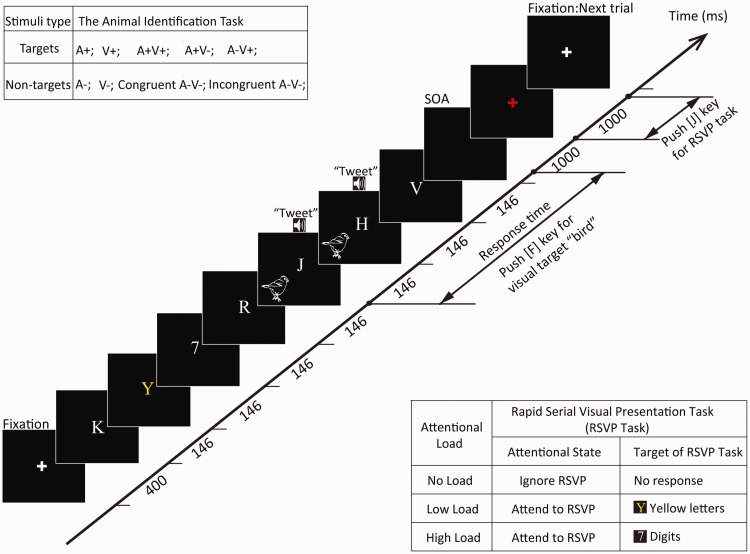
A schematic representation trial in which both the animal identification task and the RSVP task were run simultaneously. The participants must judge whether the presented animal image or sound represents a “bird” while ignoring the RSVP streams (no load), reporting yellow letters (low load), or reporting numbers (high load). Each trial began with a central fixation cross (400 ms), followed by a stream of seven characters (letters or numbers), which were sequentially presented with random replacement every 146 ms, while an animal picture or sound (300 ms) was randomly presented alongside the first to fifth letter of the RSVP streams. Participants should respond as soon as possible to the “bird” picture or sound by pressing the “F” key, and they were asked to press the “J” key for the target of the RSVP task when the red fixation point appeared (1,000 ms). RSVP = rapid serial visual presentation; SOA = stimulus onset asynchrony.

The stimuli in the RSVP task consisted of 23 distractor letters of the alphabet (A, C, D, E, F, J, H, J, K, L, M, N, P, Q, R, S, T, U, V, W, X, Y, Z) and seven digits (2, 3, 4, 5, 6, 7, 9). Some letters (I, B, O) and digits (1, 8, 0) did not appear in the RSVP streams because the visual similarity between the letters and digits could be confusing to the participants. The RSVP streams were presented continuously during the animal identification task (Figure 2). Each letter/number (each subtending 2.0° × 2.0°) in the RSVP stream was presented centrally for 146 ms.

### Design

The factorial design had two within-subject factors: stimuli type (V+, A+, Congruent A + V+, Incongruent A + V−, Incongruent A−V+) and attentional load (no load, low load, high load).

In the present study, we employed a dual-task design to explore whether semantic congruency modulates the effects of attentional load on AV integration. First, we controlled for semantic congruency in the AV integration task; the semantically congruent/incongruent stimuli comprised animal pictures presented along with either congruent or incongruent auditory stimuli. Second, we adopted the RSVP task used in Gibney et al. (2017) as the distractor task to impose different levels of attentional load as follows: no load, low load, and high load. Specifically, the participants simultaneously performed the AV integration task and a distractor task that required them to search a central RSVP stream for either a yellow letter (low load) or a white digit (high load). In addition, under the no-load condition, the participants were instructed to ignore the presented RSVP stream (Talsma et al., 2007). In addition, previous dual-task studies have used similar RSVP streams composed of letters and numbers with a colour change representing a low-load target and/or a number representing a high-load target (Gibney et al., 2017; Ho et al., 2009; Santangelo & Spence, 2007).

### Procedure

Our study included three attentional-load condition types by adopting an RSVP task, namely, no load, low load, and high load.

Under the no-load condition, although the pictures and sounds of animals were presented simultaneously with RSVP streams, participants simply needed to perform the animal identification task (participants had to judge whether the present animal image or sound is a “bird”) and were not instructed to search for the targets in the RSVP streams (Talsma et al., 2007). In our experiments, each trial began with a 400-ms presentation of the fixation cross to indicate the beginning of a new trial. An animal picture was randomly presented alongside the first through fifth letter of the RSVP stream in each trial. During the experiment, participants were instructed to make a button-press response (the “F” button on the computer keyboard) as soon as possible with their right index finger when a picture or sound target (“bird”) occurred. A blank interface (1,000 ms) was presented to ensure sufficient time to respond to the animal identification task (Figure 2).

The low-load condition consisted of the presentation of an RSVP yellow letter detection task and the animal identification task (Gibney et al., 2017; Ho et al., 2009; Santangelo & Spence, 2007). In the animal identification task, the stimuli and procedures were identical to those under the no-load condition (participants were asked to judge whether the image or sound of an animal is a “bird”), while in the RSVP task, the participants were required to detect infrequent yellow letters. Each trial began with a central fixation cross presented for 400 ms, followed by a stream of seven characters (letters or numbers), which were continuously displayed at a rate of 6 Hz. Specifically, these different letters were sequentially presented, being randomly replaced every 146 ms. This random replacement was restricted in such a way that a letter was always replaced with a different letter or digit. The target of the RSVP task was presented with equal probability in the first through seventh positions in the stream. The letters in the stream were chosen randomly prior to each trial, with the sole restriction being that no distractor was repeated within a given stream. Specifically, the RSVP streams in each trial had a 25% probability of containing no numbers or yellow letters, a yellow letter only, a number only, or a yellow letter and a number, thus resulting in a 50% probability of a target being present in each trial for all attentional-load conditions. With respect to the RSVP task, participants were asked to respond at the end of each trial, i.e., after the red fixation point (1,000 ms) appeared, subjects were asked to press the “J” button if they observed a target during the RSVP task (Figure 2).

Under the high-load condition, while the target of the RSVP task was a digit, the other requirements were the same as those under the low-load condition, notably because the task of searching for digits in a series of letters (high load) requires a higher level of semantic processing and more attentional resources than the task of searching only for a specific colour under the low-load condition (Gibney et al., 2017; Ho et al., 2009; Santangelo & Spence, 2007). In this way, by increasing the difficulty of the distractor task, we can control the attentional resource that can be used by AV integration processing.

The experiment included 4 blocks of 144 trials each under each load condition, and each block lasted approximately 7 min. Thus, it takes about 28 min for each load condition. Participants were permitted to take breaks between blocks. In addition, each load condition was completed in a separate block, and the order in which participants completed the load condition blocks was randomized and counterbalanced across participants. Before the experiment was officially started, all participants engaged in a practice experiment with 30 trials to ensure that they correctly understood the experimental procedures and responded correctly to the different tasks.

### Data Analysis

Because Bayesian analysis provides a measure of evidence regarding how much more probable the null hypothesis is compared with the alternative hypothesis (Wagenmakers et al., 2017) and does not depend on the stopping rule (Dienes, 2014; Rouder, 2014), for all tests, in addition to *p* values, Bayes factors are also reported. A Bayes factor above 3 is indicative of substantial evidence for the alternative hypothesis, whereas a Bayes factor below 1/3 indicates substantial evidence for a null hypothesis; between these values indicates the data are insensitive (Dienes, 2014). Bayes factors were calculated using a half-normal distribution. In addition, in each analysis, the degrees of freedom were corrected using the Greenhouse–Geisser correction when the Mauchly’s test indicated that the assumption of sphericity had been violated.

#### Analysis of the Influence of the Distractor Task

First, to check the RSVP performance to verify that participants accurately performed the distractor task (because they could have simply ignored it and only attended the primary task), the percentage of accuracy under different load conditions were analysed. A Shapiro–Wilk test was conducted to confirm the assumption of a normal distribution in low-load and high-load conditions. If the Shapiro–Wilk test was not significant, the repeated-measures analyses of variance (ANOVAs) for comparisons between different load conditions were conducted. If the Shapiro–Wilk test was significant, we used the one-way nonparametric repeated-measures ANOVAs (the Friedman test) for comparisons. Statistical significance was considered for *p* values < .05.

Second, we calculated the relative performance under the no-load, low-load, and high-load conditions for all stimuli (A+, V+, A + V+, A + V−, A−V+) to explore whether attentional load significantly disrupted the response times (RTs) for the AV integration task.

In addition, dual-task interference was quantified by calculating a dual-task effect (DTE) of each task (Plummer & Eskes, 2015). To test whether the load manipulation worked, we calculated the DTE of the changes in RT in the multisensory task between the dual task and single task to compare the trial types. For the variables in which higher values indicate worse performance (e.g., RT), the DTE was calculated as follows (Plummer & Eskes, 2015):

(1) $$\mathrm{DTE}\left(\%\right)=\frac{-\left(\mathrm{dual}\;\mathrm{task}\;\mathrm{RT}-\mathrm{single}\;\mathrm{task}\;\mathrm{RT}\right)}{\mathrm{single}\;\mathrm{task}\;\mathrm{RT}}\text{×}\;100\%$$

Similar measures have been used in other published dual-task paradigm studies (Gibney et al., 2017). Therefore, negative DTE values indicate that attentional load decreased performance (i.e., dual-task cost). We calculated the DTE of the changes in the RT between the no-load and low-load conditions (but not between the no-load and high-load conditions) to compare the trial types. We conducted a repeated-measure ANOVA with DTE as the dependent factor and stimuli modalities (A+, V+, A + V+, A + V−, and A−V+) as the independent factor to explore whether the attentional load has different influences on different stimuli modalities.

#### Analysis of the AV Integration Task

RTs are defined as the times between the onset of the target presentation and the behavioural response. Incorrect trials and trials with RTs shorter than 200 ms or longer than 1,200 ms were also excluded from the analysis (3.11%). Median RTs, accuracy, and response distributions for each trial type were calculated for each subject. The median RTs of each participant under each condition were used in the RT analysis as RT distributions are generally skewed and the median is less affected by the presence of outliers. Median RTs were calculated for attentional-load conditions, i.e., no load, low load, and high load, and were separated by modality, i.e., V+, A+, A + V+, A + V−, and A−V+. The main effects and interactions of load condition and modality type were analysed using repeated-measures ANOVA with 5 stimuli modalities (V+, A+, A + V+, A + V−, A−V+) × 3 attentional loads (no load, low load, high load). Percentage of accuracy was analysed by nonparametric repeated-measures ANOVAs (the Friedman test). Statistical significance was considered for *p* values < .05.

#### Calculation of Cumulative Distribution Functions

##### Race Model of Semantically Congruent AV Stimuli

To test whether participants integrated the semantically congruent AV stimuli under each load (Laurienti et al., 2006; Miller, 1982, 1986), we used the individual cumulative distribution functions (CDFs) of each target modality in each load condition to calculate the race model using the following formulas:

$$P({\mathrm{RT}}_{\mathrm{Race model}}<t)=P({\mathrm{RT}}_{A}<t)+P({\mathrm{RT}}_{V}<t)$$

This inequality does not require the channel processing times to be stochastically independent, and this prediction allows one to rule out all separate-activation models (Gondan & Minakata, 2016; Miller, 1982). Thus, it is suitable for calculating the race model inequality. In this formula, the race model provides the probability (P) of an RT that is less than a given time in milliseconds, where time ranges from 200 to 1,200 ms after stimulus onset. In addition, race model inequality violation is based on the combination of the unimodal auditory and unimodal visual CDFs (Miller, 1986). The percentiles of the semantically congruent AV CDF of each participant in each load condition were compared with the corresponding race model CDF (e.g., no-load AV CDF vs. no-load race model CDF) at each time bin to test for race model inequality violations (Gondan & Minakata, 2016; Ulrich et al., 2007). Two-tailed paired *t* tests were used to analyse race model inequality violations (Ulrich et al., 2007; Van der Stoep et al., 2017; the resulting *p* values were Bonferroni corrected; *p* < .05). Significant violations of the race model (i.e., RT_AV_ < RT_Race model_) indicate AV interactions that exceed statistical facilitation.

Because each subject has a different time course for his or her responses, averaging difference curves across individuals may not provide a complete indication of group differences (Van der Stoep et al., 2015). Moreover, in previous studies, the positive area under the difference curve was used as a measure of AV integration, and it was not affected by timing differences across individuals (Hugenschmidt et al., 2009; Stevenson et al., 2014). Thus, we specifically calculated the positive area under the difference curve (i.e., the difference in probability of the congruent AV CDF and the race model CDF for the RT range from 200 to 1,200 ms) to test for differences in race model inequality violation between different attentional loads. We also followed the approach described with RSE-box to analyse the positive area under the difference curve (Otto, 2019). To extract the positive area under the difference curve, all negative probabilities (no race model violation) were set to a value of zero, and only the positive area under the curve was calculated for all participants (Van der Stoep et al., 2015, 2017). We then compared the positive area under the difference curve between attentional-load conditions using a repeated-measure ANOVA with the factor attentional load (no load, low load, and high load) to explore how attentional loads influence the integration of semantically congruent AV stimuli.

##### Distractor Effect of Semantically Incongruent AV Stimuli

To assess the distractor effect of semantically incongruent AV stimuli, the CDFs for responses to unisensory targets were subtracted from the CDFs for responses to incongruent AV targets, yielding a relative distractor effect (Mozolic et al., 2008). Specifically, the CDFs for responses to unisensory auditory targets (A+) were subtracted from the CDFs for responses to incongruent AV targets (A + V−; auditory targets with visual distractors) to obtain a measure of the visual distractor effects for incongruent AV targets; the comparison between unisensory visual targets (V+) and incongruent AV targets (A−V+; visual targets with auditory distractors) produced the auditory distractor effect for incongruent AV targets. At each time bin, we performed two-tailed paired *t* tests to evaluate the difference in probability between unisensory CDFs and incongruent AV CDFs from 200 to 1,200 ms in each load condition to assess significant differences in the visual/auditory distractor effect in different load conditions (*p* < .05; the resulting *p* values were Bonferroni corrected).

Furthermore, we specifically calculated the negative area under the difference curve (i.e., the difference in probability of the unimodal CDF and the incongruent AV CDF for the RT range from 200 to 1,200 ms) to examine differences in the distractor effect for incongruent AV targets in different load conditions. To extract the negative area under the difference curve, all positive probabilities were set to a value of zero, and only the negative area under the curve was calculated for all participants. We determined the negative area under the difference curve by calculating the trapezoidal area between each time bin that produced a negative distractor effect. Each trapezoidal negative area between each time bin was summed to provide a total negative area for each load condition. We then compared the negative area under the difference curve between different attentional-load conditions using a repeated-measure ANOVA with the factors of attentional load (no load, low load, high load) to determine how the visual/auditory distractor effect for AV incongruent targets was influenced by attentional-load conditions.

## Results

### The Influence of the Distractor Task

First, because the Shapiro–Wilk test for the accuracy of the RSVP task under each load condition was not significant (low load: *W* = 0.948, *p* = .388; high load: *W* = 0.931, *p* = .202), we conducted the repeated-measures ANOVA to determine whether accuracy in the RSVP task was reduced by attentional load. The results indicated that the accuracy of the RSVP task was significantly higher under the low-load condition (*M* = 90.4%, *SE* = 0.79) than that under the high-load condition (*M* = 84.8%, *SE* = 1.37), *F*(1, 17) = 23.84, *p* < .001, η^2^ = 0.584, BF_(10)_= 340.0. Moreover, the accuracy of the RSVP performance was greater than 80%, indicating that the participants accurately performed the distractor task; the participants did not only perform the AV integration task under the low-load and high-load conditions.

**Figure 3.**
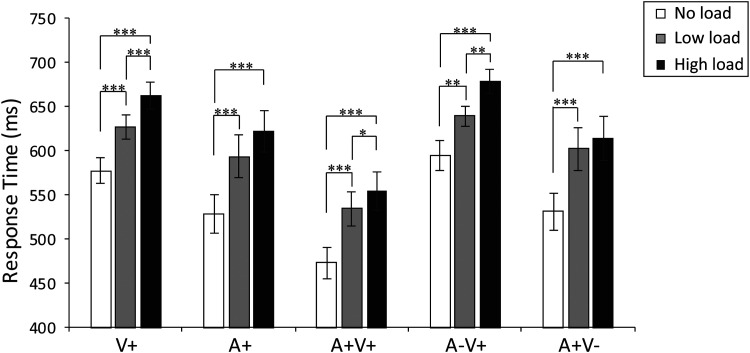
The median RTs under the unimodal (A+ and V+), bimodal congruent (A + V+), and bimodal incongruent (A−V+ and A + V−) conditions are presented under different load conditions. The response times to all stimuli generally increased as the load increased. The error bars represent the standard error of the mean. ***p < .001, **p < .01, *p < .05.

Second, the repeated-measures ANOVA using stimulus modality (V+, A+, A + V+, A−V+, A + V−) and attentional load (no load, low load, high load) as factors in the AV integration task revealed a main effect of load, *F*(2, 34) =37.744, *p* < .001, η^2^ = 0.689, BF_(10)_= 1.33 × 10^33^; the post hoc test showed that interparticipant median RTs for the AV integration task were significantly slower under the low load condition (*M* = 617, *SE* = 18) compared with the no load condition [*M* = 557, *SE* = 17, *t*(17)= 2.78, *p* = .034, BF_(10)_= 1.29 × 10^4^]; and the median RTs under the high load condition (*M* = 642, *SE* = 19) were slower than those under the low load condition, *t*(17)= 6.67, *p* < .001, BF_(10)_= 1.45 × 10^16^. These results indicated that the high-load task was more demanding. Furthermore, the attentional load significantly disrupted the RTs, regardless of the sensory modality (Figure 3), specifically for V+ stimuli [no load/low load: *t*(17) = –5.6, *p* < .001, BF_(10)_= 854.35; no load/high load: *t*(17) = –8.09, *p* < .001, BF_(10)_= 5.2 × 10^4^; low load/high load: *t*(17) = –4.71, *p* = .001, BF_(10)_= 1.27 × 10^2^]; A+ stimuli [no load/low load: *t*(17) = –6.0, *p* < .001, BF_(10)_= 1.7 × 10^3^; no load/high load: *t*(17) = –6.0, *p* < .001, BF_(10)_= 1.48 × 10^3^; low load/high load: *t*(17) = –1.46, *p* = .18, BF_(10)_= 0.558]; A + V+ stimuli [no load/low load: *t*(17) = –3.5, *p* < .001, BF_(10)_= 1.3 × 10^3^; no load/high load: *t*(17)= –6.42, *p* < .001, BF_(10)_= 3.25 × 10^3^; low load/high load: *t*(17)= –2.9, *p* = .036, BF_(10)_= 4.57]; A−V+ stimuli [no load/low load: *t*(17) = –3.5, *p* = .009, BF_(10)_=15.05; no load/high load: *t*(17) = –7.91, *p* < .001, BF_(10)_= 455.1; low load/high load: *t*(17) = –4.14, *p* = .003, BF_(10)_= 38.55]; and A + V− stimuli [no load/low load: *t*(17) = –5.0, *p* < .001, BF_(10)_= 243.3; no load/high load: *t*(17) = –4.94, *p* < .001, BF_(10)_= 185.2; low load/high load: *t*(17) = –1.08, *p* = .31, BF_(10)_= 0.389]. In summary, the RTs to all stimulus modalities (A+, V+, A + V+, A + V−, A−V+) were significantly slower under high-load than under no-load (all *F* > 1, all *p* < .01). Hence, the identification of targets in the AV integration task was slower under low-load and high-load conditions versus no-load conditions regardless of the sensory modality.

**Figure 4.**
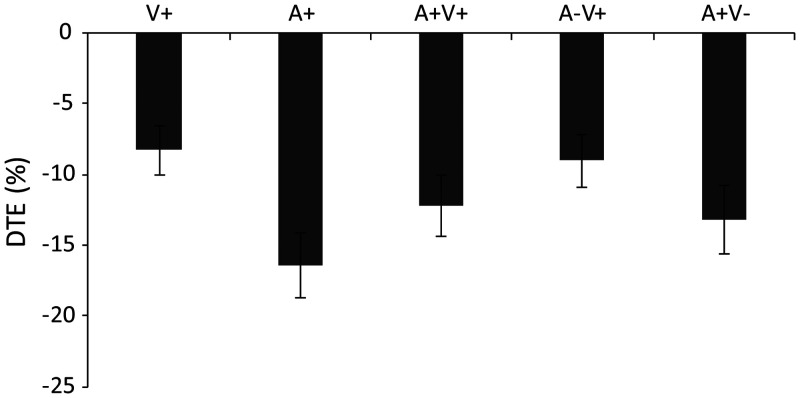
The response time DTE of different stimulus types in the identification task. The sign of the DTE for response time was reversed so that the increased response time is represented as a negative DTE. DTE = dual-task effect.

In addition, the DTE values of the RT in all trial types in the AV integration task were negative, indicating that attentional load decreased performance (Figure 4). A repeated-measures ANOVA of the five stimulus modalities (V+, A+, A + V+, A−V+, and A + V−) did not show a significant main effect of the stimulus modalities, *F*(1.96, 33.32) = 2.98, *p* = .065, η^2^ = 0.149, BF_(10)_= 2.3, suggesting that the DTEs of the changes in RT in the AV integration task did not significantly differ across the trial types.

Overall, these results demonstrated two key findings. First, we checked the RSVP performance and verified that participants accurately performed the task, and the load manipulation was indeed functional (the RSVP performance was lower under the high-load condition than the low-load condition). Second, the load manipulation indeed interfered with the target processing in the AV integration task because the RTs to all target stimuli were significantly decreased by attentional loads.

### Performance of the AV Integration Task

#### Response Times

We performed two planned comparisons to study (a) RT bimodal facilitation (A + V+ compared with A+ and V+ together) under all load conditions and (b) the distractor effect (comparison of V+ with A−V+ and A+ with A + V−) under different attentional loads (Table 1).

To determine how attentional load interacts with semantic congruency to influence AV integration, we conducted repeated-measures ANOVA on median RT using stimulus modality (V+, A+, A + V+, A−V+, A + V−) and attentional load (no load, low load, high load) as factors. Significant main effects of stimulus modality, *F*(1.382, 23.498) =44.798, *p* < .001, η^2^ = 0.725, BF_(10)_= 6.876 × 10^28^, and load, *F*(2, 34) =37.744, *p* < .001, η^2^ = 0.689, BF_(10)_= 1.33 × 10^33^, were observed. However, we did not find a significant interaction between stimulus modality and load, *F*(3.02, 51.32) = 1.789, *p* = .084, η^2^ = 0.095, BF_(10)_= 0.034. To test our main hypotheses in detail, we then analysed this result separately under different load conditions by conducting plan-tests. Post hoc subsidiary analyses with Bonferroni adjustment for multiple comparisons (plan-tests) demonstrated the following.

**Figure 5.**
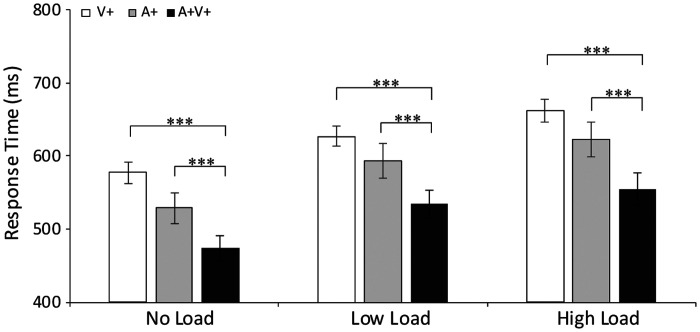
The median response times in the animal identification task. Comparison of the magnitudes of the mean response times in the unisensory visual (V+), auditory (A+), and bimodal congruent (A + V+) trials under the no-load, low-load, and high-load conditions. Error bars represent the standard errors of the means. ***p < .001, **p < .01.

**Figure 6.**
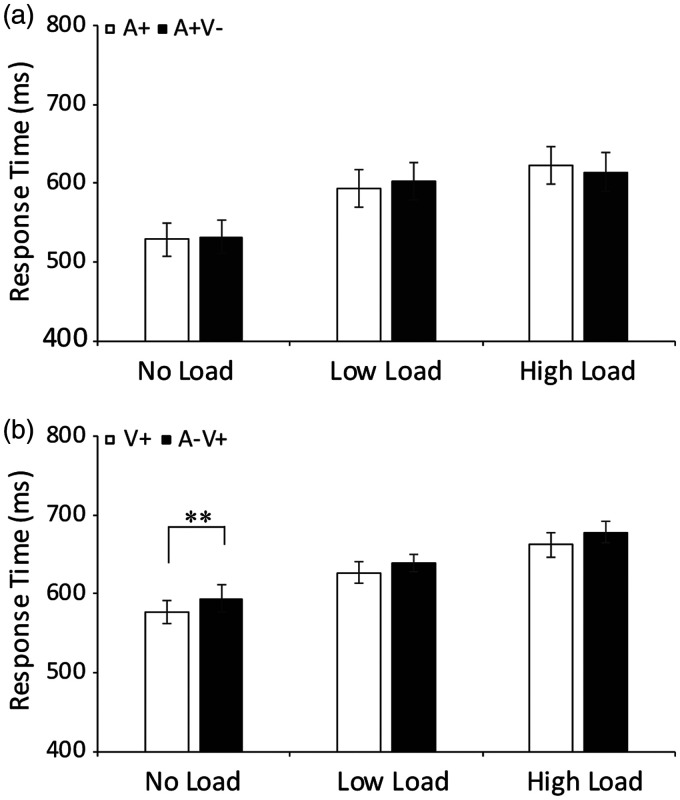
The median response times in the animal identification task are presented. (A) Comparison of the magnitudes of median response times for unimodal auditory trials (A+) and bimodal incongruent A + V− trials under no-load, low-load, and high-load conditions. (B) Comparison of the magnitude of median response times for unisensory visual trials (V+) and bimodal incongruent A−V+ trials under no-load, low-load, and high-load conditions. **p < .01.

1. The median RTs for the A + V+ trials were significantly faster than those for the V+ trials [no load: *t*(17) = 14.6, *p* < .001, BF_(10)_ = 5.25 × 10^8^; low load: *t*(17) = 10.25, *p* < .001, BF_(10)_= 8.71 × 10^5^; high load: *t*(17) = 9.67, *p* < .001, BF_(10)_= 3.08 × 10^5^]; or the A+ trials under each load condition [no load: *t*(17) = 4.5, *p* = .004, BF_(10)_= 85.28; low load: *t*(17) = 6.78, *p* < .001, BF_(10)_= 6.972 × 10^3^; high load: *t*(17) = 8.5, *p* = .006, BF_(10)_ = 1.79 × 10^5^] (Figure 5). This finding suggests that the identified speed advantage for the semantically congruent AV target over both types of unisensory targets was observed under all load conditions.

2. The median RTs for the A + V− trials were not significantly slower than those for the A+ trials under all load conditions [no load: *t*(17) = 2.25, *p* = .413, BF_(10)_= 1.676; low load: *t*(17) = 0.67, *p* = .529, BF_(10)_= 0.292; high load: *t*(17) = 1.29, *p* = .209, BF_(10)_= 0.506] (Figure 6A). In addition, the median RTs for the A−V+ trials were significantly slower than those for the V+ trials under the no-load condition, *t*(17) =3.67, *p* = .008, BF_(10)_= 43.5, but there was no significant difference under the low-load and high-load conditions[ ]low load: *t*(17) = 2.67, *p* = 0.291, BF_(10)_= 2.213; high load: *t*(17) = 1.0, *p* = .313, BF_(10)_= 0.389 (Figure 6B). This observation revealed an auditory interference effect only under the no-load condition, and attentional load hindered this distractor effect.

#### Accuracy

The accuracy in the AV integration task in all load conditions violated the Shapiro–Wilk tests (all *W* < 1, all *p* < .01), and the nonparametric Friedman tests on the accuracy of AV integration task showed significant differences under different load conditions, (χ^2^(14)=77.22, *p* < .001). The Wilcoxon signed-rank tests on the coefficient of variance showed significant influences for some stimulus types in the no-load condition [V+ vs V + A+, *W*(18)= –2.414, *p* = .016; A+ vs V + A+, *W*(18)= –2.512, *p* = .012]; low-load condition [V+ vs V + A+, *W*(18)= –2.99, *p* = .003；A+ vs V + A+, *W*(18)= –2.61, *p* = .009]; and high-load condition [V+ vs V + A+, *W*(18)= –2.95, *p* = .003；A+ vs V + A+, *W*(18)= –3.308, *p* = .001]. However, there was no significant difference for other stimulus types in the no-load condition [V+ vs V + A−, *W*(18) = –0.061, *p* = .952; A+ vs V−A+, *W*(18) = –0.71, *p* = .944]; the low-load condition [V+ vs V + A−, *W*(18) = –1.85, *p* = .065; A+ vs V−A+, *W*(18) = –0.284, *p* = .776]; and the high-load condition [V+ vs V + A−, *W*(18)= –1.51, *p* = .131; A+ vs V−A+, *W*(18)= –1.62, *p* = .106]. These results showed that although advantageous nature of A + V+ stimuli over V+ and A+ were observed under different load conditions, the distracting nature of A−V+ and A + V− stimuli was not found under all load conditions. Thus, the RT effects were not due to a speed accuracy trade-off, and based on the generally very low error rates, the distracting nature of multisensory stimuli might be reflected mostly in RTs.

#### Cumulative Distribution Functions

##### Race Model Violation of Semantically Congruent AV Stimuli

Consistent with the median RT comparisons that showed similar significant multisensory gains under all attentional-load conditions, the comparisons between the semantically congruent AV CDF and the race model CDF under each load condition for each time bin revealed significant race model inequality violations for all load conditions (*p* < .05, paired *t* test, two-tailed, Bonferroni corrected). A significant race model inequality violation was observed from 430 ms to 500 ms in the no-load condition (*p* < .05), from 410 ms to 520 ms in the low-load condition (*p* < .05), and from 480 ms to 540 ms in the high-load condition (*p* < .05). The range of RTs in which the significant race model inequality violation was observed under the no-load condition was not greater than that observed for the low-load and high-load conditions.

**Figure 7.**
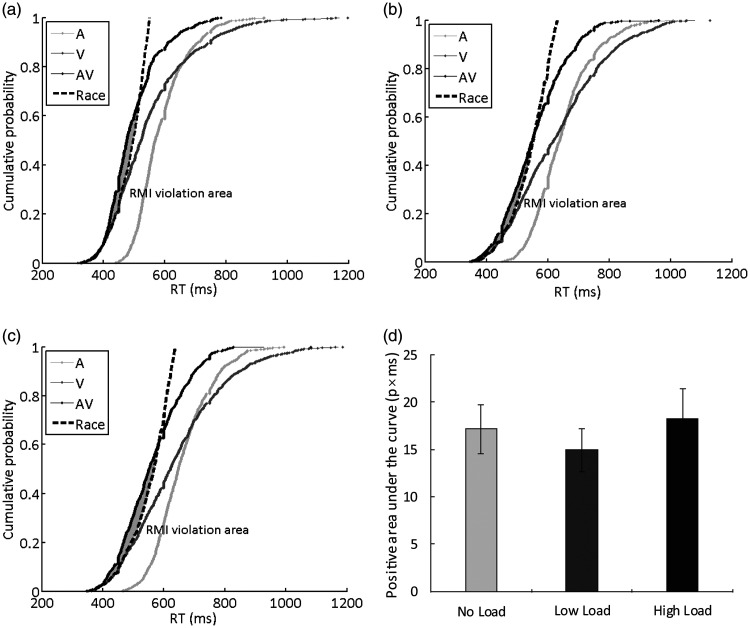
Distributions of the response times under different load conditions. (A) Cumulative distribution functions (CDFs) for the discrimination response times to auditory, visual, semantically congruent audiovisual stimuli, and race model under no-load condition. (B) CDFs under the low-load condition. (C) CDFs under the high-load condition. (D) No significant difference was observed across different load conditions for the positive area. RT = response time.

In addition, the positive area under the curve was compared between different load conditions (Figure 7). The repeated-measures ANOVA revealed that attentional load did not significantly modulate the positive area under the curve, *F*(1.747, 29.69) = 0.635, *p* = 0.517, η^2^ = 0.036, BF_(10)_ = 0.231. Notably, a Bayes factor below 1/3 indicates substantial evidence for a null hypothesis (Dienes, 2014), and hence, the Bayesian analyses of the positive area under the curve between different load conditions clearly showed evidence for no effect of attentional load on the positive area under the curve. The post hoc paired *t* tests (Bonferroni corrected) revealed that the positive area under the curve in the no-load condition (*M* = 17.17 ms, *SE* = 2.57) was also not significantly larger than that in the low-load condition [*M* = 14.94 ms, *SE*= 2.28, *t*(17) = 0.907, *p* = .377, BF_(10)_= 0.349], and high-load condition [*M* = 18.28 ms, *SE* = 3.08, *t*(17) = –0.321, *p* = .752, BF_(10)_= 0.255]; there was also no significant difference between the low-load and high-load conditions—low load/high load: *t*(17) = –1.096, *p* = .288, BF_(10)_= 0.410 (Figure 7D). These results indicated that attentional load did not affect the overall strength of semantically congruent AV integration.

##### The Interference Effect Produced by Semantically Incongruent AV Stimuli

**Figure 8.**
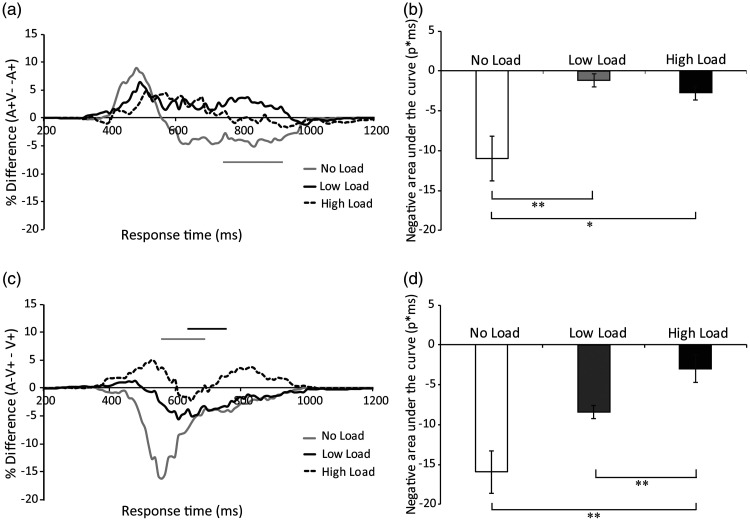
(A) Visual and auditory distractor effects under no-load, low-load, and high-load conditions. The subtraction of A+ CDF from A + V− CDF yields the visual distractor effect, but a significant visual distractor effect is only present under the no-load condition. (C) The subtraction of V+ CDF from A−V+ CDF yields the auditory distractor effect, but a significant auditory distractor effect is only present under the no-load and low-load conditions. The average negative area under the curve in each load condition was plotted separately for the visual distractor (B) and auditory distractor (D) effects, demonstrating that both were reduced by attentional load. **p < .01, *p < .05.

To assess the effects of nonmatching cross-modal distractors, we compared the response distributions for different unisensory trials with the response distributions for nonmatching multisensory trials under different attentional conditions (Figure 8).

##### Visual Distractor Effect

A comparison between the auditory (A+) CDF and semantically incongruent A + V− CDF in each time bin showed a visual distractor effect; however, this visual distractor effect was only observed under the no-load condition (*p* < .05, paired *t* tests, two-tailed, Bonferroni corrected, Figure 8A). Specifically, a visual distractor effect was observed at 770–930 ms in the no-load condition (*p* < .05), but no visual distractor effect was found under the low-load or high-load condition. The negative area under the curve was compared between the different load conditions (Figure 8B). The repeated-measures ANOVA revealed a main effect of load, *F*(1.285, 21.84) = 9.235, *p* = .004, η^2^ = 0.352, BF_(10)_= 195.3. The post hoc test revealed that the negative area under the curve of the visual distractor effect in the no-load condition (*M* = –10.95 ms, *SE* =2.8) was significantly larger compared with the low-load condition [*M* = –1.18 ms, *SE* =0.82, *t*(17) = 3.72, *p* = .005, BF_(10)_= 23.7], and high-load condition [*M* = –2.76 ms, *SE* =0.88, *t*(17) = 2.69, *p* = .046, BF_(10)_= 3.7], but there was no difference in the negative area between the low-load and high-load condition, *t*(17) = 1.2, *p* = .74, BF_(10)_= 0.45. This result suggested that attentional load reduced the visual distractor effect.

##### Auditory Distractor Effect

In addition, the comparison between visual (V+) CDF and semantically incongruent A−V+ CDF in each time bin revealed an auditory distractor effect, but this auditory distractor effect was only observed under the no-load and low-load conditions (*p* < .05, paired *t* tests, two-tailed, Bonferroni corrected, Figure 8C). Specifically, an auditory distractor effect occurred at 520–660 ms under the no-load condition and at 610–760 ms under the low-load condition; the auditory distractor effect was not found under the high-load condition. The negative area under the curve was compared between the different load conditions (Figure 8D). The repeated-measures ANOVA revealed a main effect of load, *F*(1.078,18.323) = 12.168, *p* = .002, η^2^ = 0.417, BF_(10)_= 1194.9. The post hoc test showed that the negative area of the auditory distractor effect was significantly larger in the no-load condition (*M* = –15.95 ms, *SE* =3) as compared with the high-load condition, *M* = –2.96 ms, *SE* =0.72, *t*(17) = 4.142, *p* = .002, BF_(10)_ = 52.05, but not compared with the low-load condition, *M* = –8.4 ms, *SE* =0.84, *t*(17) = 2.32, *p* = .099, BF_(10)_= 1.992. The negative area under the curve in the low-load condition was significantly larger than the high-load condition, *t*(17) = 7.39, *p* < .001, BF_(10)_ = 1.72 × 10^4^. This result suggested that attentional load reduced the auditory distractor effect.

## Discussion

The present study sought to determine how attentional load interacts with semantic congruency to influence the AV integration of common objects. We used an RSVP task to manipulate the amount of attentional resources that were available for the integration processing of semantically congruent and incongruent animal sounds and images. Our results revealed that attentional load did not eliminate the AV integration of semantically congruent animal sounds and images (Figures 5 and 7). However, semantically incongruent AV stimuli were not integrated (as there was no multisensory facilitation) under all load conditions, and attentional load attenuated the multisensory interference effect produced by semantically incongruent animal sounds and images (Figures 6 and 8). The integration of semantically congruent AV object features appeared to be more robust to attentional load manipulation than the multisensory interference effect of semantically incongruent AV object features. Thus, our finding provides evidence that semantic congruency modulates the effect of attentional load on the AV integration of common objects.

To the best of our knowledge, the present study is the first to demonstrate that attentional load does not eliminate the AV integration of semantically congruent animal sounds and images (Figures 5 and 7). Regarding the facilitation effect produced by semantically congruent bimodal stimuli, it has been proposed that relevant semantic unimodal information could be rapidly integrated into a coherent multisensory representation (i.e., within 100 ms in certain cases; Giard & Peronnet, 1999; Lehmann & Murray, 2005; Murray et al., 2004; Shams et al., 2005) and that the effective mental representation formed by semantically congruent AV stimulus can be well matched with the inherent characteristics already present in memory systems (Laurienti et al., 2004; Molholm et al., 2007; Stein & Meredith, 1993); thus, the consolidation and integration processing of semantically congruent AV information is enhanced. One possibility that could explain why the integration of semantically congruent AV object features can resist external interference is related to the “attentional load theory,” which postulates that tasks involving a high perceptual load that requires full capacity leave little capacity for the processing of irrelevant distractor information (Lavie, 2005; p. 1). However, as the goal of the present study involves identifying a visually presented animal image (or identify an animal sound), the presentation of a semantically congruent animal sound (or a congruent animal image) could provide coherent and useful information for identification of the target; furthermore, most task-relevant inputs can be prioritized given that they are highly relevant to the current task. Therefore, it is difficult for attentional load to hinder the integration of semantically congruent AV object features.

It is noteworthy that one neuroimaging study has explained why multisensory cues retain their ability to capture a participant’s attention, even under conditions of attentional or memory load (Zimmer & Macaluso, 2007; see Spence & Santangelo, 2009 for a review). Specifically, the multisensory control may still mediate modulatory effects from higher order frontoparietal regions even when there is a uncoupling between cross-modal effects in the visual cortex and working memory/sustained visuospatial attention such that multisensory interactions between visual-tactile stimuli seem to be relatively unaffected by manipulations of visual load (Zimmer & Macaluso, 2007). Similarly, it has been proposed that a multisensory or supramodal cortical region higher in the information processing hierarchy (e.g., polysensory superior temporal sulcus) might send signals to the unisensory cortices to modulate the processing of the features of common objects, even when some features of a particular object are not explicitly attended (Eimer et al., 2002; Molholm, 2007), because the neural representations of features of common objects are likely to be strongly and tightly bound together (Amedi et al., 2005; Beauchamp et al., 2004). This phenomenon may indicate that higher order multisensory cortical regions can still play an important mediating role in the multisensory interaction between semantically congruent AV features of common objects, even without much attentional resources. Moreover, when the time period between prime-target pairs that share a semantic relationship is shorter than 200 ms, the semantic priming processing for prime-target pairs of the same object is relatively automatic (Sachs et al., 2008). Therefore, the integration of semantically congruent AV object features can also occur even when attentional resources are exhausted.

Nevertheless, attentional load has a different effect on the multisensory interference effect produced by semantically incongruent AV object features. Consistent with previous findings (Mozolic et al., 2008; Suied et al., 2009), we observed an auditory distractor effect (incongruent A−V+ compared with unimodal V+) and a visual distractor effect (incongruent A + V− compared with unimodal A+) under the no-load condition (see Figure 8A and C), but these interference effects of the semantically incongruent animal sounds and images were attenuated by attentional load (Figure 8B and D). It is possible that if the presented AV stimuli are semantically incongruent, the mismatch between the actual sensory input and prediction in the memory system could lead to a major update of the internal model of the mental representation (Klemen & Chambers, 2012; Talsma, 2015); in such a case, the presence of semantically incongruent AV objects could cause a certain degree of an interference effect and impair behavioural performance. Furthermore, the brain does not absorb the mismatched auditory information into the memory system (i.e., it should be rapidly forgotten) if the presented sound is not semantically consistent with the representation of the target images because this incongruent information is useless in the relevant task (Chen & Spence, 2010; Potter, 1999). Thus, under the conditions of limited and absent attentional resources, the top-down modulatory mechanism underlying selective attention processes may automatically filter task-irrelevant mismatched information, further preventing irrelevant stimuli from entering the memory system, increasing the speed of the forgetting process and resulting in reduced interference effects.

We further observed an asymmetric cross-modal interference effect supporting the visual dominance hypothesis; specifically, the auditory distractor effect (unimodal V+ compared with incongruent A−V+) was stronger than the visual distractor effect (unimodal A+ compared with incongruent A + V−) under all attentional-load conditions (see Figures 6 and 8). When no attentional load is added, it has been proposed that the different interference effects produced by semantically incongruent AV (A + V−, A−V+) stimuli may occur because the attention system itself is not completely supramodal (Alais et al., 2006; Keitel et al., 2013); in other words, attentional modulation of sensory neural processing in the visual cortex can occur at least partially independently from similar attentional modulations to auditory processing (Talsma et al., 2006), and a possible asymmetry may exist in the attentional filtering of irrelevant auditory and visual information (Suied et al., 2009). Therefore, during the processing of semantically incongruent AV stimuli, the ability to filter irrelevant visual distractors is stronger compared with irrelevant auditory distractors, resulting in the auditory distractor effect (unimodal A+ compared with incongruent A + V−) which is stronger than the visual distractor effect (unimodal A+ compared with incongruent A + V−). Notably, one possibility to consider regarding the asymmetric cross-modal interference effect under increased load conditions is, because studies investigating object-based attention tasks across sensory modalities suggest that attentional resources are at least partially distinct for the visual and auditory sensory modalities (Alais et al., 2006; Keitel et al., 2013; Talsma et al., 2006), and the presence of a visual RSVP stream might make participants to focus strongly on the visual modality and occupy a large amount of visual attentional resources, more attentional resources can remain to process task-irrelevant auditory distractors than irrelevant visual distractors. Thus, the auditory distractor effect (A−V+ compared with unimodal V+) will be stronger than the visual distractor effect (A + V− compared with unimodal A+) even under low-load and high-load conditions.

One could argue that the RSVP task applied herein (low load vs. high load) is not a load manipulation but rather a task switch (colour vs. digit detection task) that interferes with AV integration. We think this possibility exists, as this switch between two tasks in response mappings does cause some interference. Notably, the colour or digit detection task inevitably consumes certain attentional resources and competes for the cognitive resources of AV integration task given that the accuracy of the RSVP task was greater than 90%, and the performance on the RSVP task decreased as the load increased. Furthermore, the levels of the load manipulation (colour vs. digit detection task) tap into the same type of processing resources because the detection of colours and digits belongs to object recognition (the so-called what; Chan & Newell, 2008; Wahn & König, 2017), and notably, the task of searching for digits in a series of letters (high load) requires a higher level of semantic processing and more attentional resources than the task of searching only for a specific colour under the low-load condition.

Furthermore, one could also argue that attentional load manipulation (by adopting RSVP tasks) may only interfere with processing in the visual sensory modality (in the AV integration task) but has no effect on processing in the auditory sensory modality. Indeed, when applying the dual-task methodology, a general concern is whether the two tasks compete for the same pool of attentional resources or whether multiple resource pools are used to separately address the various cognitive and perceptual aspects of the two tasks (Wahn & König, 2017). In fact, some researchers have proposed that the recruitment of shared or distinct attentional resources across sensory modalities is partially task-dependent (Chan & Newell, 2008; Wahn & König, 2015, 2016) and depends on whether the tasks involve object-based attention (e.g., colour or shape), spatial attention (e.g., localization of stimuli), or both (Wahn & König, 2017). In addition, it has been proposed that in the visual and auditory sensory modalities, if object-based attention tasks are time-critical, shared resources are recruited across the sensory modalities (Hunt & Kingstone, 2004; Marois & Ivanoff, 2005; Wahn & König, 2017). Because the main task we adopted is an object recognition task and the distractor task (RSVP task) involving searching for either a yellow letter or a white digit is also an object attention task, we considered the RSVP tasks to interfere with target processing in both sensory modalities in a previous study. Moreover, our results showed that the RSVP task not only interfered with target processing in the visual sensory modality but also significantly interfered with target processing in the auditory sensory modality (Figures 3 and 4), further confirming that the RSVP tasks we adopted interfered with target processing in both sensory modalities.

Of note, if we choose other alternative tasks as distractor tasks in future research, we may obtain different experimental findings. For example, it has been proposed that when an object-based attention task is performed along with a spatial attention task, distinct attentional resources are required for the auditory and visual sensory modalities if a visual attentional load is induced (Arrighi et al., 2011; Wahn & König, 2017). Therefore, if a visuospatial task (i.e., a multiple object tracking task) was adopted as the visual distractor task, it selectively interfered with the visual discrimination task while the auditory discrimination performance was not affected. Furthermore, a question worthy of further investigation is whether multisensory integration can still occur even if the load task is multisensory.

## Conclusion

The experiments described herein indicate that semantic congruency modulates the effect of attentional load on the AV integration of common objects. Specifically, the performance enhancements associated with semantically congruent AV object features are present even when attentional resources are limited; however, semantically incongruent animal sounds and images were not integrated, and attentional loads influenced the multisensory interference effect produced by incongruent AV object features.

**Table 1.** Median Accuracy (%) and Response Times (RTs, ms) With Standard Deviations (SDs) for Each Trial Type Under No-Load, Low-Load, and High-Load Conditions.

	No load		Low load		High load	
	RTs (*SD*)	Accuracy (*SD*)	RTs (*SD*)	Accuracy (*SD*)	RTs (*SD*)	Accuracy (*SD*)
V+	577.2 (61.2)	99.0 (1.4)	626.9 (56.6)	96.2 (4.1)	662.2 (63.6)	96.1 (5.9)
A+	528.3 (91.5)	97.3 (4.6)	593.4 (102.5)	95.1 (7.0)	622.2 (101.8)	92.5 (9.2)
A + V+	473.2 (76.3)	99.9 (0.4)	534.5 (81.7)	93.3 (1.4)	554.7 (93.7)	98.9 (3.0)
A−V+	593.9 (72.2)	98.9 (2.1)	639.4 (48.6)	98.0 (3.2)	678.4 (59.3)	98.4 (3.3)
A + V−	531.2 (88.7)	97.5 (4.0)	602.1 (102.0)	95.1 (5.4)	613.9 (103.6)	95.1 (6.9)
